# Elevated breast cancer risk among mothers of a population-based series of 2668 children with cancer

**DOI:** 10.3332/ecancer.2008.57

**Published:** 2008-01-17

**Authors:** D Pang, G Evans, J Birch

**Affiliations:** 1 Cancer Research UK Paediatric and Familial Cancer Research Group, Royal Manchester Children’s Hospital; 2 Department of Medical Genetics, St Mary’s Hospital, University of Manchester, Manchester, UK

## Abstract

**Aims:**

Although a previous study found high risk of breast cancer in mothers of children with soft tissue sarcomas, breast cancer risks in mothers of sufferers of other childhood cancers largely remain unknown. The aetiology is not fully understood. The present study explored this excess by varying type of childhood solid cancer and formulated a hypothesis.

**Methods:**

Mothers of 2668 children with solid tumours included in the Manchester Children’s Tumour Registry, 1954–96, were traced and followed up to 31 December 2000 through the UK National Health Service Central Register. Standardized incidence ratio (SIR), p-values and 95% confidence intervals were calculated from age and calendar-year-specific female breast cancer incidence rates for England and Wales.

**Results:**

There was a significant excess of breast cancer in mothers overall (SIR=1.3, 95%CI=1.0–1.5) mainly due to mothers of children with rhabdomyosarcoma (RMS) (SIR=2.2, 95%CI=1.0–4.0), skin cancer (SIR=7.9, 95%CI=2.9–17.1) and central nervous system tumours (SIR=1.2, 95%CI=0.9–1.8). Maternal breast cancer risk was associated with late age at birth of the index child, and male sex and young age at diagnosis in the index child. Risk was highest in the ten years, following the birth of the index. The pattern was seen most strongly in mothers of children with embryonal RMS.

**Conclusion:**

There are excesses of breast cancer in mothers of children with solid tumours in general and specifically in RMS, skin and central nervous system (CNS). There appears to be a temporal relationship between certain tumours in children and breast cancer in their mothers, suggesting an origin of their respective pregnancy. We propose a mother–foetal interaction mechanism to explain this association.

## Introduction

Since a twofold increased risk of breast cancers among first degree relatives of children with soft tissue sarcomas was reported [1, 2], it has been found that there is an increased breast cancer risk among the mothers of children with solid tumours in general [3] and specifically with soft tissue sarcoma [4,5]. The breast cancer risks of mothers in other solid tumours have not been explored. A possible mechanism largely remain unknown although it is known that germline mutations in certain cancer-associated genes, such as the TP53 or BRCA2 gene, predispose to the development of both specific childhood cancers, for example sarcomas or CNS and adult breast cancer [6–10]. The purpose of the present study was to investigate this excess by analysing risks of maternal breast cancer associated with various types of solid tumour. We also sought to describe this association by characteristics in children and mothers and attempted to formulate a hypothesis to explain the pattern of breast cancer risk observed in the mothers.

## Materials and methods

### Study subjects

The mothers of 2668 children with histologically confirmed solid tumours diagnosed younger than 15 years of age, who were included in the Manchester Children’s Tumour Registry (MCTR) between 1 January 1954 and 31 December 1996 were eligible. The ascertainment procedure and the follow-up procedure have been described in detail elsewhere [3]. A summary is provided below. Following approval from the appropriate research ethics committees, mothers of the index children were traced and followed up to 31 December 2000 through the UK National Health Service Central Register (NHSCR), providing continuous notification of details of cancer registrations and causes of death (flagging). Diagnosis of carcinoma of the breast was coded according to the contemporaneous revision of the International Classification of Diseases for Oncology [11, 12] and the International Classification of Diseases. All diagnoses in children were coded according to International Classification of Diseases for Oncology Second Edition and regrouped into a smaller number of diagnostic categories according to the Birch and Kelsey (BK) classification scheme, which groups biologically similar tumours together [13]. All cases were categorized on the basis of morphology and topography codes into broad groupings as follows: Hodgkin disease (HD), non-Hodgkin lymphoma (NHL), central nervous system (CNS) tumours, which included benign and borderline tumours, retinoblastoma (RB), neuroblastoma, peripheral primitive neuroectodermal tumours (PPNET), Wilms tumour (WT), hepatoblastoma (HB), osteosarcoma and chondrosarcoma (Osteo & Chondro), rhabdomyosarcoma (RMS), other bone and soft tissue (ST) sarcoma, gonadal germ cell tumours (GCT), non-gonadal GCT, adrenocortical carcinoma (ACC), malignant neoplasms of skin (melanoma and non-melanoma), carcinoma (excluding of skin), other rare specified tumours, and unspecified neoplasms. The morphology and topography code allocations defining BK groups and algorithms for selecting the groups are available upon request.

### Statistical methods

Expected numbers of breast cancers were calculated from serial age-specific female breast cancer incidence for England and Wales, from 1971 onwards, applied to the similarly defined arrays of person-years at risk generated by the data. National rates were used rather than north-west England regional rates to allow for migration of families out of the region during the lengthy follow-up period. All population data were supplied by the Office for National Statistics, London, UK. For earlier years at risk, the incidence rates for 1971 were used to calculate expected numbers. To calculate the risk of carcinoma of the breast, mothers entered the person-years at risk on the date of birth in the index child and left the person-years at risk on the date of diagnosis of carcinoma of the breast, date last known to be alive, date of embarkation from the UK, date of death, or the closing date of the study, whichever was the earlier. Two-sided p-values and 95% confidence intervals (CI) of standardized incidence ratios (SIRs) were computed on the assumption the observed numbers followed a Poisson distribution. All analyses have been carried out using Stata [14].

Separate analyses were initially performed for groups of mothers defined by diagnostic group in the index child. In those groups, showing a statistically (or marginally) significantly elevated SIR, the excesses were explored in more detail by comparing sub-groups defined by characteristics in the children and their mothers according to the following rationale. Germline mutations may lead to earlier ages of cancer onset. We hypothesized that early age of diagnosis in the index child may be a marker for breast cancer risk in the mothers. We therefore analysed breast cancer risks in selected sub-groups of mothers whose children were below the population median age for diagnosis of their respective tumours. Similarly, we conducted analyses of breast cancers by age at diagnosis in the mothers. Factors that may indicate mother–foetal interaction in pregnancy include differences in breast cancer risks in mothers of boys compared with girls [5] and a temporal relationship between birth of the index child and onset of breast cancer in the mother. To investigate this possibility, we performed separate analyses in mothers of boys and mothers of girls and estimated breast cancer risks by time elapsed since the birth of the index child (<10 years and ≥10 years). To examine the effect of maternal age at the birth of the index, we also performed separate analyses for maternal age groups (<35 years and ≥35 years).

## Results

The mothers of 2345 (88%) were successfully traced and flagged at NHSCR. The reasons for non-trace were due to lack of name/date of birth, adoption, emigration, in prison and in the armed forces.

Table 1 shows SIRs for breast carcinoma in mothers of children with solid tumours by diagnostic groups in children. As previously reported, overall, there was a significant excess of carcinoma of the breast in the mothers (O 99, SIR 1.3, p<0.05) [3]. For individual diagnostic groups in children, significant excesses were shown among mothers of children with RMS (O 10, SIR 2.2, p<0.05), and mothers of children with malignant neoplasms of skin (O 6, SIR 7.9, p<0.001). An excess of borderline significance was found among mothers of children with CNS tumours (O 32, SIR 1.2, 95%CI 0.9–1.8). No group showed a significant deficit of breast cancer.

There was no significant excess risk of carcinoma of the breast in mothers of children with osteosarcoma and chondrosarcoma. Therefore, previous findings based on an earlier series from the MCTR were not substantiated in this extended series [15].

In view of our previous findings, mothers of children with embryonal RMS (eRMS) were analysed separately and showed a significant excess of breast cancer (O 6, SIR 2.7, p<0.05).

The following diagnostic groups were selected for sub-group analyses of maternal breast cancer risk: eRMS, skin cancer and CNS tumours (Table 2).

For the individual diagnostic group of eRMS in the index, there were significant excesses of carcinoma of the breast in mothers of boys (O 6, SIR 4.2, p<0.01), breast cancer diagnosed under 50 years old (O 4, SIR 4.7, p<0.05), breast cancer in the ten years following birth of the index (O 4, SIR 19.5, p<0.001), and in mothers above 35 years old at birth of the index (O 4, SIR 12.6, p<0.001). In addition analyses by age at diagnosis in the index child showed that mothers of children under median age at diagnosis had a much higher SIR of 7.3 (O 6, p<0.001) than mothers of children at the median age and above (SIR=0). This was due to mothers of children in the first age quartile at diagnosis (O 5, SIR 14.4, p<0.001). For skin cancer in the index child, there were significant excesses among mothers in all sub-groups. However, markedly higher SIRs were observed for mothers in the first ten years, following the birth of the index and aged 35 years or more at the birth of the index. The population median age at diagnosis of skin cancer is above 60 years. Therefore, no analyses by age of the index case were carried out since all index cases were exceptionally young and located at the extreme lower end of the age distribution. When melanoma and non-melanoma skin cancers were analysed separately, a similar pattern of results was obtained for both groups. For individual diagnostic group of CNS tumours in the index, non-significant SIRs above 1.5 were found for mothers of boys, mothers in the ten years following birth of the index, and mothers above 35 years old at birth of the index. PNET was the only CNS tumour sub-group with a population median age below 15. Analyses of breast cancer in mothers of children with PNET diagnosed below the median age of nine years showed O 8, SIR 1.9 and 95%CI 0.8–3.8.

## Discussion

The study indicates that the excess risk of carcinoma of the breast among mothers of children with solid tumours is not uniform across all groups, but is associated with a small number of tumour types and patient characteristics.

The observed pattern is seen most strongly in eRMS. There are higher risks in mothers of children with eRMS diagnosed at very young ages, mothers of boys and in the ten years following birth of the index, suggesting an origin of the respective pregnancy. A biologically feasible interpretation would be hormonal mother–foetal interaction in pregnancy in genetically susceptible individuals [5].

This is suggested by higher risks of developing carcinoma of the breast in mothers in the first few years after birth of the index and higher risk in mothers of boys. It is likely that in these children the eRMS originates before birth. It is known that the foetus itself is actively involved in the production and regulation of oestrogen, a hormone that is an established aetiological factor in breast cancer.

Risk factors for breast cancer, identified in population-based studies, include early menarche, late menopause, late first full-term pregnancy, and use of exogenous oestrogens. All these factors increase the amount of oestrogen to which the breast epithelium is exposed [16]. To meet the demand for oestrogen, during pregnancy foetal adrenals, grow rapidly and disproportionately [17]. In the ten weeks between 20th and 30th week of pregnancy, the foetal adrenal cortex undergoes a high rate of cell proliferation and the size and weight of foetal adrenals are doubled, reaching 10–20 times the relative size of the adult adrenals. Enormous quantities of C19 steroid (DHEA-S), 100–200 mg/day are produced by the foetal adrenal cortex for oestrogen synthesis.

The foetal adrenal cortex also undergoes a process of maturation involving apoptosis (programmed cell death), a basic process in tissue remodelling, to eventually produce the mature adrenal cortex. Apoptosis starts in the 14th week, increases with advancing gestation and is maximal during the first postnatal month [18].

We hypothesize that a germline defect in the foetus in a gene involved in cell-cycle control, apoptosis and tissue development, results in abnormal levels of steroid hormones via aberrant functioning of the foetal adrenal cortex, due to failure of the normal process of apoptosis and maturation.

This would explain the observed temporal relationship between pregnancy, birth of the index child, development of tumour in the child and development of breast cancer in the mother. One such gene is TP53.

The differences in risks between mothers of boys and girls with eRMS might suggest that the relevant cancer-associated genes in male foetuses are more important in maintaining development and normal functioning of fast growing organs, such as the foetal adrenal cortex than in females. Male foetuses grow faster and males have higher average birth weight but shorter gestations than girls [19, 20].

The striking effects of maternal age at birth of the index child may suggest that timing of pregnancy in mothers is also important. In older women, there may be a greater chance that precancerous changes are present in the breast epithelium. In these circumstances, carrying a foetus with a cancer predisposing mutation may trigger the process of carcinogenesis, and thus accelerate the development of breast cancer, following the birth of the index child. The effect would be even more marked in mothers who carried the same mutation.

A practical implication of our model is that the highest risks and earliest ages at onset would be seen in mothers of babies who were mutation carriers and who themselves also carried the mutation. These mothers are predisposed and are also subjected to abnormal exposure to steroids produced by the foetal adrenal cortex, which had failed to mature (apoptosis) in the normal way. Mothers of babies who were mutation carriers would also be at increased risk even though they themselves do not carry the mutation. We would predict that carrier mothers of non-carrier babies and non-parous carriers would have a later age of onset (though still young) than carrier mothers of carrier babies.

In this series of eRMS, there was one known case with a germline TP53 mutation, whose mother was also a mutation carrier. In addition, this case had a sister, 15 months older, who carried the mutation. The mother’s breast cancer was diagnosed at age 27 years, 19 months after the birth of the child. Four children with other tumours, ACC, astrocytoma and PNET (2 cases) carried germline TP53 mutations. Their mothers, who were also confirmed mutation carriers, all developed breast cancer seven years or less after the birth of the respective children.

The striking excesses of breast cancer in mothers of children with eRMS in the ten years following birth of the index are unlikely to be completely explained by a transient increase in risk of breast cancer following the first pregnancy in mothers. First pregnancy has been reported to be associated with increased risk of breast cancer in the 15 years or less after birth [21, 22]. However, the risk of breast cancer in the five years after the first birth is only just above 1 (RR=1.1) compared with nulliparous women [22]. Although highest transient increases in risk of breast cancer have been found in mothers with family history of breast cancer (RR=1.5, 95% CI 0.9–2.4) [22] and mothers above 35 years old (1.26, 95% CI 1.10–1.44) [21], the relative risks were no more than 2. For mothers of children with eRMS, the SIR was 19.5 in the ten years following the birth of the index child. Data on age at first birth in mothers of children with RMS are available since these were collected as part of more general family studies. These allow us to derive years since the birth of the first child. We carried out further analyses adjusting for maternal age at first birth (under 35 years and 35 years and above) and years since the first birth (below 15 years and 15 years and above) among the mothers, using Poisson regression, incorporating population breast cancer incidence rates [23]. The adjusted SIR is 14.7, 95% CI 4.0–54.

The observed breast cancer excess is not explained by these potential confounders relevant to the first birth. The results need not be seen as contradictory to the previous findings of transient increase in risks of breast cancer in the first pregnancy since the present study shows that the pregnancy is a crucial period in the development of breast cancer in women. However for the mothers in the present study, the pregnancy with the child who subsequently develops cancer appears to be of greater importance in the development of breast cancer than that with the first child and the striking effects in the mothers observed in the present study might reflect a different aetiology from mothers of children without cancer. The mechanism proposed above, which relates breast cancer risk to peculiarities of adrenal cortex development, might explain the marked excess.

Because of the nature of this cancer-registry-based study, retrospective direct measurement of oestrogen levels in pregnancy with the index is not possible. Similarly, access to birth records which may contain other relevant details would require individual written consent, which would be impractical since the births date back to 1939 and many index patients and their mothers have died. Furthermore, it is likely that many records will have been destroyed. However, data on birth weight are available for children with RMS as part of previous family studies. Oestrogens are known to stimulate cell proliferation and represent an important determinant of foetal growth [24]. Birthweight has been used as a surrogate measure for steroid hormone exposure during pregnancy, and a positive correlation between steroid hormone concentrations and birthweights has been observed [25].

We compared the birthweights of the children with RMS whose mothers developed breast cancer, and index children whose mothers did not, with that of live-born children in the general population. Mean birthweights of index children of mothers with breast cancer and mothers without are 257 and 54 g more than that of the general population, respectively, (with breast cancer=3600 g, without breast cancer=3397 g, population=3343 g). Mean birthweights of boys with RMS whose mothers did or did not develop breast cancer are 114 and 27g more than that of male babies in the general population, respectively. Mean birthweights of girls with RMS whose mothers did or did not develop breast cancer are 658 and 75 g more than that in the general population, respectively. There is a trend of decreasing birthweight among index cases whose mothers had breast cancer, index cases whose mothers did not have breast cancer and children in the population. This is consistent with the role of oestrogen in foetal development, and with our hypothesis relating to failure of normal maturation of the foetal adrenal cortex.

Interestingly, the present study reported an excess of carcinoma of the breast in mothers of children with skin cancer (melanoma and non-melanoma combined) with the highest excess in the first ten years, following birth of the index (SIR 23.5). Skin cancer is typically an adult-onset cancer and is very rare in children. The median age for sporadic melanoma is 67 and 35 years for familial melanoma. Therefore, all childhood cases are unusual and may be associated with genetic susceptibility. It is known that germline mutations in CDKN2A are the most common cause of inherited susceptibility to melanoma. CDKN2A encodes two proteins via alternate reading frames (ARFs). The p14ARF product, acting via the p53 pathway, can induce cell-cycle arrest or apoptosis. In multi-case melanoma families with mutations affecting p14ARF specifically there appears to be increased risk of breast cancer [26]. This is consistent with the mechanism proposed above and might explain the pattern of development of breast cancer in their mothers.

In mothers of children with CNS tumours, the present study showed borderline significant excesses among mothers of boys and mothers diagnosed in the ten years following the birth of the index. This might suggest roles of BRCA2 as well as TP53. Biallelic BRCA2 germline mutation carriers do develop childhood brain cancers as recently reported [6, 10], although BRCA2 heterozygotes do not [27]. This could be an explanation for the combination of breast cancer in a mother and brain tumour in her child. It has been reported that loss of BRCA gene function activates a p53-dependent cellular response [28], suggesting mutation in p53 may be required before inactivation of BRCA2 may lead to the development of breast cancer.

In contrast to an earlier study [15], we did not find a statistically significant increase in breast cancer risk in mothers of children with osteosarcoma and chondrosarcoma (SIR 1, 95%CI 0.6, 3.2). The SIR for breast cancer in mothers of children with retinoblastoma was of a similar magnitude (SIR 1.7, 95% CI 0.6, 4.0). We considered the possibility that there may be an excess risk of breast cancer in mothers in both groups, during early years of follow-up, mediated by mutations in the RB1 gene. However, only one mother in both groups combined was diagnosed with breast cancer in the first ten years, following the diagnosis in her child.

Considering childhood cancers are rare events, the sample size in our study is relatively large and allows us to carry out stratified statistical analyses. However, non-statistically significant results in certain diagnostic groups in children cannot completely rule out the possibility of increased breast cancer risk due to the aforementioned mechanism. Among other diagnostic groups, the highest point estimate of SIR for breast cancer is in mothers of children with adrenocortical carcinoma (ACC) although statistically not significant due to the small number. Of 12 mothers of children with ACC, two mothers developed breast cancer following birth of the child, compared with an expected number of 0.3. It is thought that ACC originates in the foetal adrenal cortex, that is an abnormality in the process of cell proliferation and programmed cell death during adrenal development. Furthermore, germline mutations in TP53 have been found among the majority of ACC cases in a number of studies [29, 30].

In this population-based study, a near complete ascertainment of children with solid tumours in a well-defined geographical area was achieved, and the case children included in the study were representative of all the case children in the corresponding population series. Therefore, the present study is unlikely to be subject to ascertainment bias due to notification of severely affected families or the knowledge of family history of cancer in surviving cases only. Neither can the excesses be explained by the bias due to the follow-up procedure, because the majority of mothers were successfully traced and there are no reasons to believe that the causes of non-trace are influenced by occurrence of breast cancer.

The study is free of reporting bias because all data were obtained from population registries rather than self-reports, that is both breast cancer in mothers and childhood cancers were derived from population-based data. However, another possible bias worth mentioning is the ‘healthy mother effect’, by analogy with the healthy worker effect, that is mothers may experience a lower breast cancer risk than those non-parous women in the general population.

Childbearing has been demonstrated to be associated with decreased breast cancer risk in human and animal studies due, in part, to the pregnancy-specific hormone, chorionic gonadotropin (hCG) [31]. The confounding effect due to childbearing in mothers in the study population, is not negligible because childless women up to 40 years old in the reference population used to estimate expected numbers represent about 20%; therefore, true SIRs would be expected to be higher than those reported here. Furthermore, there was a slightly increasing trend of breast cancer incidence in the general population since 1971. The use of the 1971 rates to calculate expected number of breast cancers before 1971 would overestimate the expected numbers. In addition, use of national rates rather than regional would also tend to overestimate the expected numbers of cases since rates tend to be a little higher in the south of England than in the north. Thus, the standardized registration ratio for breast cancer in the north-west in 2000 was 98% compared with 109% in the south-west [32]. The SIRs reported here are therefore conservative estimates of true SIRs.

## Conclusion

In conclusion, the study confirms and extends the previous findings of a temporal relationship between embryonal RMS in children and breast cancer in their mothers. We propose a hypothesis to explain these observations and suggest a possible mechanism. This mechanism may also be operating in mothers of children with other types of cancers. The present study points to pregnancy as the critical period, when mothers and children are physiologically linked. Germline mutations to cancer-associated genes, inherited or de novo might play an important role. Germline mutations might commonly target the p53 pathway affecting tissue remodelling in the foetal adrenal cortex, not just a single gene within that pathway. The pathway may be disrupted by a germline mutation within the TP53 gene itself, as well as p14ARF, BRCA1 and BRCA2, etc. Further studies of aetiology of breast cancer in mothers of children with cancer incorporating genetic/molecular measurements including mutation screening of cancer-associated genes will be useful in exploring this hypothesis. This is feasible as tissue banks and appropriate genetic techniques become available. The study may help to define periods of highest risk to enable health interventions aimed at reducing mortality from breast cancer in susceptible women.

## Figures and Tables

**Table 1:**
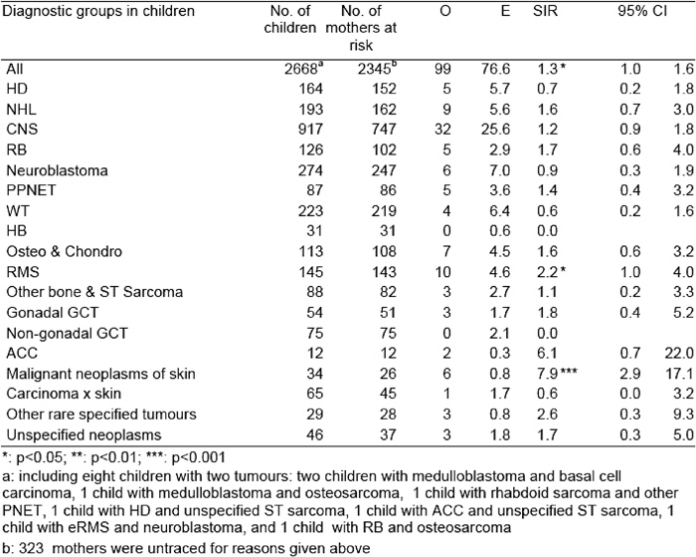
Standardized incidence ratios in mothers by diagnostic groups in children

**Table 2:**
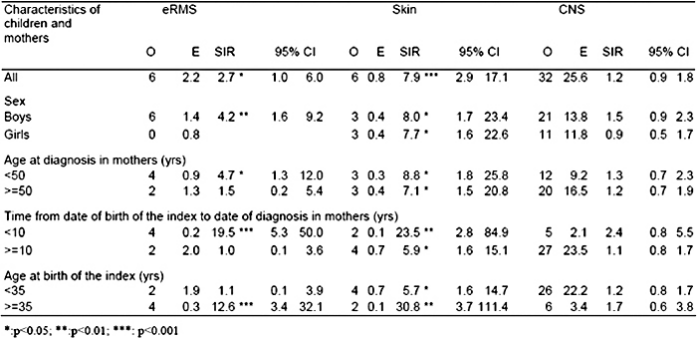
SIRs of carcinoma of the breast in mothers of children with eRMS, skin cancer and CNS tumours
